# Entropy, Free Volume, and Cooperative Relaxation

**DOI:** 10.6028/jres.102.017

**Published:** 1997

**Authors:** Shiro Matsuoka

**Affiliations:** Polytechnic University, Brooklyn, NY 11201

**Keywords:** entropy, free volume, glass transition, intermolecular cooperativity, polymer, relaxation

## Abstract

The high frequency end of the relaxation spectrum for polymer molecules involves the rotation of the segmental bonds. This fast relaxation process, however, cannot take place easily in the condensed state crowded by the densely packed conformers, necessitating the slower cooperatively synchronous relaxation. As the temperature is lowered, the domain of cooperativity grows towards the infinite size at the Kauzmann zero entropy temperature, though actually the system deviates from the equilibrium as the glass transition intervenes typically at 50 K above that temperature. The excess enthalpy and entropy drop faster than predicted by the rotational isomeric states which would reach zero only at 0 K. The real Δ*C*_P_ is greater than that of the RIS value. The actual volume in excess of the crystalline lattice volume, however, points towards zero at 0 K. Thus, a polymer with higher *T*_g_ typically exhibits a lower density and modulus in the glassy state. Since the configurational entropy associated with the free volume is proportional to the logarithm of the latter, the Kauzmann temperature can be scaled by ln *M*, where *M* is the algebraic average of the conformer molecular weight. The temperature dependence of the most dominant, i.e., the largest equilibrium domain size will result in the Adam-Gibbs and Vogel equations for the characteristic relaxation time. The cooperative domain distribution leads to the relaxation spectrum that follows a power law. The relationship between the characteristic relaxation time and the rate of physical aging is derived.

## 1. Introduction

Is it the relaxation or retardation spectrum that would better reflect molecular motions?

There are two clearly different types of viscoelastic experiments. One is to control the strain (deformation) and the other is to control the stress. Data of the first type include the relaxation modulus *G*(*t*), the secant modulus under the constant rate of strain, the dynamic modulus G*(*ω*), and the dynamic viscosity η*(*ω*). The relaxation spectrum describes the intensity of each mode of relaxation with a unique rate of dissipating the energy. It is considered to be proportional to the number of relaxing units. The strain energy is closely related to the change of molecular configuration in polymers.

The scheme of relating the relaxation spectrum to molecular relaxation seems justified by the apparent success of the Takayanagi model [1] for predicting the dynamic moduli for heterogeneous structures, such as composites, block copolymers and semicrystalline polymers, from the moduli of individual components weighted according to their volume fractions.

The second type, or the stress-controlled type, includes the creep compliance *J*(*t*), the secant compliance under the constant rate of stress increase, the dynamic compliance J*(*ω*), and the dynamic fluidity ϕ*(*ω*). Unlike the strain energy, a new approach will have to be devised which will relate the stress energy to molecular strains, and this has not been done. More assumptions are needed to interpret a retardation spectrum in molecular terms than a relaxation spectrum. However, these data can be converted to the first type mathematically.

Because the dynamic compliance J*(*ω*) is simply the reciprocal of the dynamic modulus G*(*ω*) in one and the same experiment, J*(*ω*) can be included in the first group, provided the data are analyzed in terms of the relaxation, not retardation, spectrum, and its significance can be analyzed in molecular terms.

In linear viscoelasticity, the creep compliance *J*(*t*) is not simply the reciprocal of the relaxation modulus *G*(*t*), but it can be exactly related to *G*(*t*) by the equation below from the fact that the complex dynamic modulus G* = *G*′ + *iG*″ is the reciprocal of the complex dynamic compliance J* = *J*′−*iJ*″:

$$\int_{0}^{t}G\left(t-x\right)J\left(x\right)dx=t$$ (1)

together with three other similar convolution integrals. If the relaxation modulus can in fact be represented by the sum of *G_i_* exp(−*t*/*τ_i_*) terms, then it can be seen that the creep compliance cannot be in the form of the sum of the *J_i_*(1−exp(−*t*/*τ_i_*)) terms. All models utilizing any numbers of springs and dashpots fail to satisfy Eq. (1). Springs and dashpots are not suitable in describing linear viscoelastic behavior of a given material. If the rate process is assumed for the decay of strain energy under the constant macroscopic strain, the relaxation modulus can be the sum of *G_i_*exp(−*t*/*τ_i_*) terms, and all other viscoelastic functions must be consistent with this assumption. In such a case the relaxation spectrum, but not the retardation spectrum, would reflect the structure-property relationship.

The relaxation spectrum *H*(ln) is approximately:

$$H\left(\ln\tau \right)=-\frac{dG\left(t\right)}{d\ln t},\;\tau =t.$$ (2)

It will be shown later that the intensity of relaxation *H*(ln*τ*) is proportional to the number of participating conformers, and *H*(ln*τ*) ≅ *G*″(ln*ω*), particularly when |dlog*G*″/dlog*ω*|<<1.

The relaxation spectrum may be “normalized” by the absolute value of the modulus, and

$$\frac{H\left(\ln\tau \right)}{G*\left(\omega \right)}\approx \frac{G^{\prime \prime }\left(\omega \right)}{G*\left(\omega \right)}=\frac{J^{\prime \prime }\left(\omega \right)}{J*\left(\omega \right)}.$$ (3)

This quantity is approximately equal to the slope of the double logarithmic relaxation modulus curve when G*(*ω*) ≅ *G*(*t*), such as in the solid-like state. The dielectric loss factor *ε*″(*ω*) is similar to *J*″(*ω*), and it too can be a basis of molecular interpretation.

## 2. The Conformer

Theodorou and Suter [2] have demonstrated with computer simulation that an overwhelming proportion of elastic deformation in amorphous polymers is taken up by partial rotation of the main chain bonds. This is not a surprising conclusion, and it can be carried further by suggesting that the irreversible bond rotation from one stable configuration to another in the presence of a force field is the basic mode of relaxation. The rotation of one single bond from a gauche to a trans configuration is the fastest mode of relaxation involving the smallest unit, called conformer. In a vinyl polymer, a monomeric repeat unit consists of at least two conformers.

When a conformer changes the configuration, it must pass an intermediate stage that has a much higher free energy level, amounting to 12.6 kJ/mol to 15.1 kJ/mol of conformer. If molecular weight of the conformer is 50 and the density is 1 g/cm^3^, this means 3 × 10^9^ ergs, which is 10 times greater than the strain energy spent at the yield for a typical glassy polymer. Since the mechanically imposed strain energy is so small as compared to this barrier energy, the probability for a successful rotation, i.e., relaxation, can be considered as controlled by the probability of these thermally excited conformers reaching this energy barrier. It becomes the bottle neck for the irreversible transition in configuration, and its relative population determines the rate constant for the process. The relative population is proportional to the exponential function of negative energy difference over *kT*, according to the Maxwell-Boltzmann equipartition principle, so that the rate of transition is obtained from the equation:

$$p\propto \exp\left(-\frac{\Delta \mu }{kT}\right),$$ (4)

where *p* is the probability that the gauche configuration will spontaneously transit to the trans state, and Δ*μ* is the highest free energy level the bond angle must pass, relative to the stable state in equilibrium. *k* is Boltzmann’s constant and *T* is the absolute temperature. As is well known, this is the classical theory of rate processes [3], and the rate constant is proportional to the probability *p*. The time constant for this transition is the relaxation time for a conformer and it is proportional to *p*^−1^, or exp(Δ*μ*/*kT*).

This relaxation time is of the order of 10^−10^ s at room temperature [4], and with Δ*μ* of 12.6~15.1 kJ, it is estimated to be 10^−12^ s at 500 °C. This, then, comprises the shortest time end of the vast spectrum of many modes of relaxation a polymer molecule is capable of undergoing. The dominant “characteristic relaxation time” of polymers is many orders of magnitude slower even at 50 K above *T*_g_. In the dense liquid with crowded conformers, relaxation requires a simultaneous rotation among several neighboring molecules, and this can increase the relaxation time by many orders of magnitude. Such cooperativity is not unique to polymers, but general to most glass-forming liquids.

Polymers are unique, on the other hand, in their capability for exhibiting an extremely slow relaxation process well above their glass transition temperature, and this behavior too has been described by the same word “cooperativity.” The context for this latter usage is different from the former, as the latter involves a large overall configurational change of strings of conformers which make up a polymer chain. Their individual motions add up to a net movement of a large segment of a chain. Coordinated motions among strings or “strands” of a polymer molecule can be slower by orders of magnitude than the cooperative relaxation among the individual conformers described above, though all relaxation modes in the latter context are coupled to ones in the former context. The former governs the time-temperature shift of the entire spectrum including the latter. The elastic modulus for the latter arises from a decrease in molecular conformational entropy accompanying the deformation, a typical rubber, whereas the elastic modulus for the former arises from the increase in internal energy, a typical solid.

## 3. Intermolecular Cooperativity

Our discussion will be limited to the “cooperativity” in the first context, i.e., the relaxation process in which a number of conformers relax simultaneously. At a high enough temperature, the volume is expanded to allow most conformers to relax individually. In the actual polymers this may be well above the degradation temperature, but the extrapolated value will be an important parameter. This temperature is defined as T*. At this temperature, the frequency and the magnitude of oscillation are such that a change to a new bond angle can be reversed in negligible time, and the theoretical meaning of the relaxation process as we understand it, begins to be lost above T*. We arrived at the value of 500 °C for T* after analyzing data for more than a hundred polymers [1].

If conformers were able to rotate without being interfered with by their neighbors, the activation energy barrier would remain constant at Δ*μ*, ca. 12.6 kJ, and the temperature dependence of the relaxation time *τ* (which is very short) would follow the Arrhenius relationship:

$$\ln\tau =\ln\tau *+\frac{\Delta \mu }{kT}-\frac{\Delta \mu }{kT*},$$ (5)

where τ* denotes the relaxation time at T*.

Because of the neighboring interference, however, the relaxation time grows at a faster rate with temperature change than shown by Eq. (5). The degree of cooperativity is characterized by defining the domains of cooperativity, in which all conformers must relax simultaneously. The domain size, *z*, then becomes the exponent for the probability for simultaneous relaxation of otherwise independent conformers. We now obtain, instead of Eq. (5):

$$\ln\tau =\ln\tau *+\frac{\Delta \mu z}{kT}-\frac{\Delta }{kT*}.$$ (6)

Equation 6 is no longer an Arrhenius equation because *z* changes with the temperature. The concept of the temperature dependent domain size, *z*, is the center piece of the theory by Adam and Gibbs [5], although their further treatment of it seems to imply the intramolecular, rather than intermolecular, cooperativity.

The property of *z* is such that *z* = 1 at T*, and *z*→∞ at *T*_0_, where the domain size has grown so large that all conformers are locked together. Such a temperature dependence of *z* can be met with the equation:

$$z=\frac{T}{T-T_{0}}\cdot \frac{T*-T_{0}}{T*}.$$ (7)

A real liquid vitrifies at about 50 K above *T*_0_, but amorphous glassy polymers are far from the state of zero configurational entropy [6]. Extrapolation from above *T*_g_ to 50 K below *T*_g_ will hardly increase the molecular order, and it is safe to say that configurational entropy is unlikely to extrapolate to 0 at *T*_0_. The configurational entropy *S*_c_ should be estimated from the statistics of the rotational isomeric state, and this entropy would reach 0 at 0 K. So, what kind of entropy is it that Kauzmann [7] observed heading toward 0 at *T*_0_? To clarify this question, one needs to examine how the configurational entropy per mole of conformer changes as the domain size grows.

The configurational entropy *S*_c_ is calculated from the concentration dependence of the gauche and trans configurations, where the concentration *ϕ* for the gauche state depends on the difference in the energy level from the trans state, *ΔΓ*:

ϕ=2exp(−ΔΓ/kT)/[1+2exp(−ΔΓ/kT)]. (8)

The factor 2 is placed in the formula because there are three possible angles that a conformer can assume per bond. In some polymers, the third position is forbidden by the geometrical interference; in such a case this factor 2 is replaced by unity. The entropy of mixing between the trans and gauche configurations is obtained through an approximation:

$$S_{c}≅-6k\left[\phi \ln\phi +\left(1+\phi \right)\ln\left(1-\phi \right)\right].$$ (9)

The factor 6 is placed in the equation because each of the two conformers (trans or gauche) has a choice of three bond angles.

**Fig. 1:**
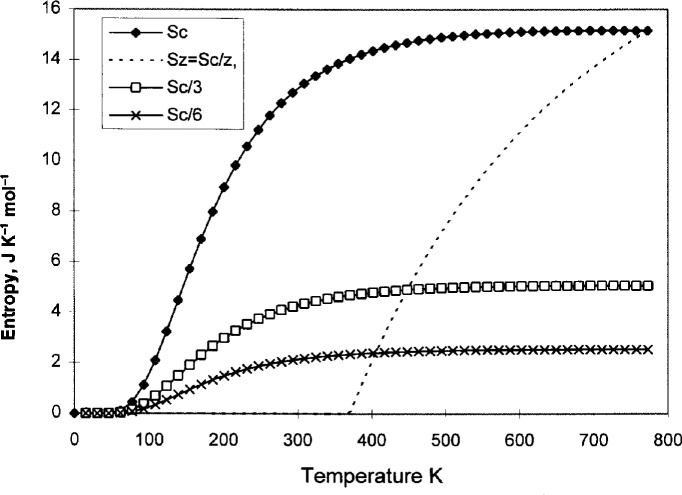
The conformational entropy per conformers, per domains of size *z* = 3, *z* = 6, and *z* that depends on temperature (the dotted line). *T*_0_ = 370 K is chosen. (Note that the units of entropy are in moles of conformer.)

Now, in a domain that consists of *z* conformers, all configurational changes must occur together, and that means the degree of freedom inside the domain is the same as that of one free conformer. The configurational entropy *S*_c_ is reduced to *S*_z_ = *S*_c_/*z* because of the cooperative restrictions. In other words, the entropy *S*_c_ is reduced at all temperatures but still headed toward zero at 0 K, while *S_z_* = *S*_c_/*z* decreases rapidly toward zero at *T*_0_. This is illustrated in Fig. 1, showing *S*_c_, *S*_c_/3, *S*_c_/6, and *S_z_*.

**Fig. 2:**
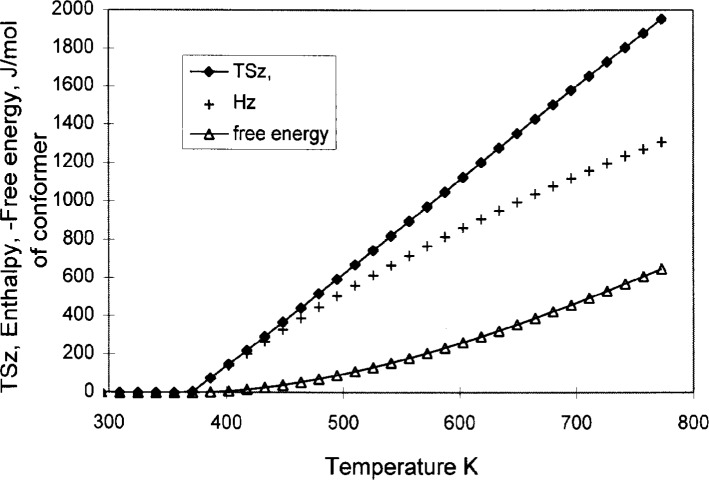
Enthalpy H_z_, the product *TS*_z_, and the Gibbs free energy (negative value), all per mole of conformers, calculated from the *S*_z_ = *S*_c_/*z*(*T*) shown in Fig. 1.

Although the celebrated theory of Gibbs and DiMarzio [8] is a theory that leads to zero entropy at *T*_0_, it is different from the cooperative domain model presented here. Gibbs and DiMarzio modified Flory’s lattice model, but by including holes that are temperature dependent, the conformational entropy is made to reach zero at *T*_0_. Still, their theory requires that polymer molecules be sufficiently ordered near *T*_0_, whereas with the model presented here the configurational entropy is reduced by virtue of the increasing cell size of the microstates as the domain grows at lower temperatures. Since the glass transition is not unique to polymers, a model must be easily adaptable to nonpolymers. The Gibbs free energy can be calculated from the temperature dependence of *S_z_*, and from it the enthalpy *H_z_* can be, also. Figure 2 is an illustration of the temperature dependence of *TS_z_*, *H_z_*, and the (negative) Gibbs free energy; *H_z_* < *TS_z_* for *T*_0_ < *T* < T*. The plot of *TS_z_* is nearly a straight line starting at zero at *T*_0_, which is useful in scaling among polymers with different values of *T*_0_.

**Fig. 3:**
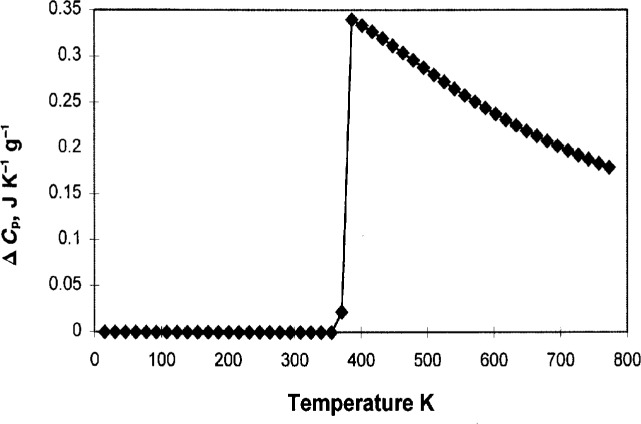
The specific heat Δ*C*_P_ per gram has been calculated since the molecular weight of the conformer can be calculated from *T*_0_. This result agrees with the data for polycarbonate.

The specific heat, Δ*C*_p_, i.e., the difference between the liquid and the solid (glass) state, is of interest, and is shown in Fig. 3, together with that which corresponds to the *S*_c_. Both values are per mole of conformers, and they must be divided by the molecular weight of conformer to obtain the value per g. The peak for d*H*_c_/d*T* appears as the consequence of the increased order at lower temperature, and the greater the energy difference Δ*Γ* between the trans and the gauche configuration, the greater this peak appearing at a higher temperature, but this should not be confused with the glass transition.

Δ*C*_p_ depends on temperature more strongly than Δ*C*_p_ ∝ 1/*T*, a prediction by Scherer [9]. It will be shown later that the specific heat per mole is proportional to the ratio T*/(T*−*T*_0_), and to ln*M*, where *M* is the molecular weight of a conformer. Δ*C*_p_ per mole of conformer is thus greater for a polymer with a larger conformer, which has a higher *T*_g_, but *C*_p_ per gram is smaller for the same with the larger conformer, following the formula (ln*M*)/*M*, with higher *T*_g_.

The relationship between *S_z_* and *S*_c_ can also be found parallel to nonpolymeric liquids that are capable of exhibiting internal configurational changes, known as Kneser liquids [10]. Domains of cooperativity among the molecules can be considered, within which the molecular relaxation can only occur together simultaneously. By the same token, *S_z_* = *S*_c_/*z* decreases toward zero at *T*_0_. Herzfeld and Litovitz [11] find the absorption of sound waves in these Kneser liquids to be many times greater than the estimated “classical” values. A sound wave consists of the adiabatic compression and expansion, and dissipation of free energy occurs when heat conduction irreversibly equalizes the temperature differences between the crest and the trough.

The “fragile liquids,” according to the well-known classification of liquids by Angell [12], are also those which can exhibit internal degrees of freedom when free from the neighboring interference. In almost all “fragile” cases, |d*z*/d*T*| is large, i.e., *T*_0_ is closer to T*, and T*/(T*−*T*_0_) is large, and the relaxation time follows the Vogel equation. The other class of liquids termed “strong” by Angell is presumably that for which the domain size either does not grow or grows little over the temperature span, and typically exhibit Arrhenius behavior. A “strong” glass has less free volume than a “fragile” one.

Substituting the temperature dependent *z*, (Eq. 7), into the Adam-Gibbs equation [Eq. (6)] will immediately give the Vogel equation [13]:

$$\ln\tau =\ln\tau *+\frac{\Delta \mu *}{k\left(T-T_{0}\right)}-\frac{\Delta \mu *}{k\left(T*-T_{0}\right)}$$ (10)

where the relationship between and Δ*μ* and Δμ* are related by the formula:

$$\Delta \mu =\Delta \mu *\frac{T*}{T*-T_{0}}.$$ (11)

The ratio T*/(T*−*T*_0_) above has appeared already, and is an important empirical material parameter. Recalling that the product *TS_z_* in Fig. 2 is a straight line which reaches zero at *T*_0_, the ratio {*TS*_C_/*TS_z_*} = T*/(T*−*T*_0_) can be scaled for all materials with different *T*_0_ values. The physical significance of the ratio T*/(T*−*T*_0_) is the conversion of the configurational probability from without intermolecular cooperativity to with intermolecular cooperativity. All thermodynamic extensive quantities are scaled by this ratio, including Δ*C*_p_ and free energy such as Δ*μ*. The true rotational barrier of a bond is Δμ* when it is free from the neighbor interference, hence Δμ* is a universal constant for conformers that involve a C−C bond rotation with the value of a little more than 12.6 kJ/mol. Conveniently, Δμ* for paraphenylene linkage has about the same value. The ether linkage, on the other hand, has only 4.8 kJ, so an adjustment should be made when estimating *T*_g_ from polymers that include it in the repeat unit, as will be discussed later.

The Vogel equation, Eq. (10), is exactly the same as the Williams-Landel-Ferry equation [14] with appropriate substitution of the parameters, and also the same as the Doolittle free volume Eq. (15) when the thermal expansion coefficient of the fractional free volume (an empirical parameter different from the van der Waals free volume) is equated to *k*/Δμ* in the Vogel equation. This will be shown later. For the “universal” WLF equation, *c*_2_^g^= *T*_g_ − *T*_0_ ≈ 50 K, and *c*_1_^g^ = 17.4 [16]. The latter is related to the universal Δμ*, while the former is an indication that a typical glass transition occurs at 50 K above *T*_0_. All these equations pertain to the condition for thermodynamic equilibrium, i.e., above *T*_g_.

To summarize the relationship among these quantities and limiting temperatures, the following equation may be written:

$$z=\frac{S_{c}}{S_{z}}=\frac{TS_{c}}{TS_{z}}=\frac{T*-T_{0}}{T*}\frac{T}{T-T_{0}}.$$ (12)

Substitution of Eq. (12) into the Adam-Gibbs equation, Eq. (6), results in another, more widely known form of the Adam-Gibbs formula:

$$\ln\tau =\ln\tau *+\frac{\Delta \mu }{kT}\cdot \frac{S_{c}}{S_{z}}-\frac{\Delta \mu }{kT*}.$$ (13)

## 4. Free Volume and Entropy

Equation (10) is a special Vogel equation for the shift factor from T*. If *T*_g_ is chosen instead of T* as the reference temperature, Eq. (10) is changed to:

$$\ln\tau =\ln\tau_{g}+\frac{\Delta \mu *}{k\left(T-T_{0}\right)}-\frac{\Delta \mu *}{k\left(T_{g}-T_{0}\right)}.$$ (14)

The above equation can be written in the form of the Doolittle’s free volume equation,

$$\ln\tau =\ln\tau_{g}+\frac{1}{\alpha_{f}\left(T-T_{0}\right)}-\frac{1}{\alpha_{f}\left(T_{g}-T_{0}\right)}$$ (15)

where *α_f_* is the thermal expansion coefficient of the free volume fraction *f* = (*V* − *V*_0_)/*V*, where *V*_0_ is the “occupied volume” *V* at *T*_0_.

We have already pointed out that *α_f_* is really a parameter, *k*/Δμ*, arising from the energy barrier for bond rotation, Δμ*. The smaller the Δμ*, e.g., for the ether linkage, the greater the empirical *α_f_*, irrespective of the thermal coefficient for the van der Waals (real) free volume, Δ*α*, the difference in *α* between the liquid and the solid (a glass or the crystal). Whereas the Doolittle free volume vanishes at *T*_0_ like *S_z_*, the “real” free volume vanishes at 0 K like *S*_c_. In order to be consistent, the following relationship should hold:

$$\frac{\Delta \alpha }{\alpha_{f}}=\frac{T*-T_{0}}{T_{0}}$$ (16)

and here again we find the familiar ratio. The empirical value for *α_f_* ~ *R*/Δμ* obtained from viscoelastic data is about 6 × 10^−4^ K^−1^ if Δμ* is a little more than 12.6 kJ, whereas the values of Δ*α* and *α_f_* for many amorphous polymers vary around 4 and 6 × 10^−4^ K^−1^, respectively [16]. The ratio Δ*α*/*α_f_* ought to be 0.5 if *T*_g_ = 150 °C, and 0.85 for *T*_g_ = −110 °C. For polyethylene, taking *α* for the crystalline lattice for *α* of the solid, the value for Δ*α* = *α*1−*α*_s_ is 5.7 × 10^−4^ K^−1^ [17]. Furthermore, if one assumes Δμ* to be 16.7 kJ, Doolittle’s *f*_g_ of 0.025 is obtained.

The “real” van der Waals free volume with the thermal expansion coefficient of ca. 6 × 10^−4^ K^−1^ will attain 50 % of the van der Waals volume at T*. Thus a true excess volume is comparable to the true occupied volume of a conformer in the state free from the neighbors. A greater amount of free volume will be trapped in the glassy state if the *T*_g_ is higher, as has been found for crosslinked polymers with different degrees of crosslinking density [18]. Such a trapped free volume is a genuine unoccupied site that can be observed by the positron annihilation analysis. As a rule, the glassy modulus of a higher *T*_g_ polymer tends to exhibit a lower value, which is further evidence for the less dense glassy state for the high *T*_g_ polymers [19].

Now, the configurational entropy *S_f_* of the molar free volume *V_f_* can be considered to be equal to the configurational entropy of the ideal gas with the molar volume of *V_f_*, i.e.,

$$S_{f}=-k\ln\;V_{f}.$$ (17)

The absolute value of the molar free volume scales with molecular weight of conformer *M* divided by the density. Since the value of the excess entropy at T* is the same for all polymers, the product (T* − *T*_0_)ln *V_f_* is constant for all polymers. When polymers with different conformer sizes are compared, their values of T*−*T*_0_ are inversely proportional to ln(*M*/*ρ*), where *M* is the molecular weight of the conformer and is the density extrapolated to T*, or

$$\frac{T*}{T*-T_{0}}\propto \frac{\ln M}{\rho }.$$ (18)

Here again, we find the same ratio T*/(T* − *T*_0_) for scaling the extensive quantity.

Ignoring the density variations among the amorphous polymers, (in comparison to the molecular weight variations among different conformers), the constant *C* = (T* − *T*_0_) ln *M* has been evaluated through the introduction of a virtual polymer with molecular weight of *M*_0_, such that its *T*_0_ is at 0 K, i.e., *z* = 1 and *S_z_* = *S*_c_ for all temperatures from 0 K to T*. We obtain the formula:

$$\left(T*-T_{0}\right)\ln M=T*\ln M_{0}=C.$$ (19)

**Fig. 4:**
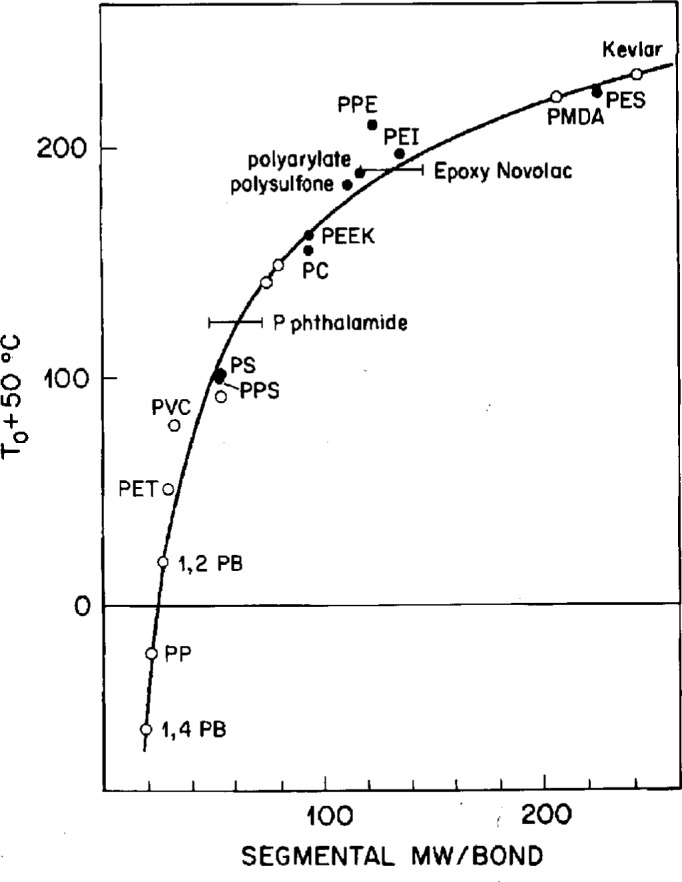
The glass transition temperature, *T*_g_ = *T*_0_ + 50 °C, vs segmental or conformer molecular weight, according to Eq. (19).

This relationship is shown in Fig. 4.

The empirically derived value for *C* is 1750 for many polymers, which means *M*_0_ = 9.6, too small for a methylene unit and not even as large as one carbon atom. For the methylene unit as a conformer, *T*_g_ of −110 °C is obtained from Eq. (19). Because this is near the *γ* transition temperature of branched (low density) polyethylene, it is often regarded as the same *β*-type relaxation. Their temperature coefficients are substantially different [20], suggesting that the glass transition for the linear polyethylene and the *γ* transition for the branched polyethylene overlap in the kHz range. The *γ* transition for branched polyethylene is definitely a local relaxation process involving a fixed number of intramolecular cooperative conformers, exhibiting a constant activation energy, just as in the case of the *β* transition in amorphous homopolymers, as will be discussed subsequently.

The above discussion, including tables of *T*_g_ for a hundred polymers, has been included in Chap. 2 of Ref. [20], but it has been further elaborated here to make the present discussion complete. Equation (19) is useful in predicting the *T*_0_, and therefore *T*_g_ ~ *T*_0_ + 50 K from the chemical structure of an amorphous polymer. The general rules for estimating the conformer size from chemical structure is elaborated below:

1. A conformer with a branch.
  - When another conformer is attached as a branch to a conformer in the main chain, the new combination attains the combined size as one conformer. When a methyl group is attached to a carbon in the main chain, this combined conformer consists of CHCH_3_ and its molecular weight is 28. However, polypropylene has a methylene unit as the other conformer along the main chain. Averaging the two, (28 + 14)/2 = 21 is obtained, and *T*_0_ of −75 °C, or *T*_g_ of −25°C is obtained from Eq. (19).
  - When the branch contains more than one conformer so the branch itself can undergo its own configurational relaxation, the number of conformers per monomeric unit increases and the average conformer size decreases again. For polyvinyl acetate, the monomeric unit consists of three conformers instead of two. The average conformer molecular weight *M* is 86/3 = 28.7, and from Eq. (19)*T*_g_ of 29 °C is obtained. Whether a further addition of conformers will raise or reduce the *T*_g_ depends on the size of a conformer to be added. Simply, if it is larger than the present average size, the *T*_g_ will rise, but if smaller *T*_g_ will fall.
2. The end group in a polymer chain has a higher enthalpy and entropy than a similar conformer inside the chain. The end group lowers *T*_g_ as would the solvent molecules. The contribution of the chain ends to the overall enthalpy can be estimated by mixing them with those conformers along the chain, and the average *T*_g_ can be obtained.
3. When compatible polymers are mixed, a value of *T*_g_ between the two individual values is found. One approach for estimating the *T*_g_ from the individual concentrations of the two polymers is to invoke the principle of isentropic state at *T*_g_ = *T*_0_ + 50 K. The entropy *S*_g_ for cooperative relaxation determines the domain size *z* at *T*_g_, so the same value for *S*_g_ means the same *z* value at *T*_g_ for all polymers and their mixtures. The entropy *S*_g_ can be derived by the formula:
  $$S_{g}≅\frac{dS_{z}}{dT}\left(T_{g}-T_{0}\right)$$ (20)
  but *T*_g_ − *T*_0_ is 50 K, and d*S*/d*T* at *T*_g_ is Δ*C*_p_ per mole of conformer, so we have:
  $$S_{g}\approx \frac{50\Delta C_{p}}{T_{g}}$$ (21)
  and Δ *C*_p_ per mole is proportional to T*/(T*−*T*_0_) or to ln*M*, hence
  $$S_{g}\propto \frac{\ln M}{T_{g}}$$ (22)
  is obtained. A mixture containing *x* mole fraction of conformer A and 1 − *x* mole fraction of conformer B will exhibit the average *T*_g_:
  $$\frac{\ln M}{T_{g}}=x\frac{\ln M_{A}}{T_{\text{gA}}}+\left(1-x\right)\frac{\ln M_{B}}{T_{\text{gB}}}$$ (23)
  which reduces to the Fox-Flory formula [21] when the variations in ln*M* are negligible.

## 5. The Relaxation Spectrum

The discussion so far has been with a single value of *z* that depended on the temperature. There no doubt is, however, a dynamic distribution of domain sizes at any temperature. The domain size as discussed up to this point is the most dominant size, which from this point on will be called the “characteristic size” *z*_c_, and the characteristic relaxation time *τ*c corresponds to this *z*_c_. *τ_c_* is the relaxation time in the Kohlrausch-Williams-Watts formula [22], exp{− (*t*/*τ*)*^β^*}. In the high temperature range, e.g., more than 100 K above *T*_g_, *z*_c_ is small and the spectrum is narrow. At moderate temperatures, e.g., 50 ~ 100 K above *T*_g_, the spectrum broadens and the Cole-Cole parameter *β* [23] is typically 0.6 to 0.9. At even lower temperatures (but in equilibrium above *T*_g_), the spectrum broadens particularly on the high frequency side, signifying the increase in domains that are smaller than *z*_c_, primarily because *z*_c_ has become larger. Cole-Davidson [24], KWW, and Havriliak-Negami [25] formulas are all for “nonsymmetrical” distributions around the characteristic relaxation time for the loss peak. (Empirically, Ngai’s [26] stretched exponential function is equivalent to the KWW formula.) When plotted in a doubly logarithmic scale, all three becomes a straight line in the high frequency side, while only H-N gives a straight line on the low frequency side with an additional adjustable parameter. Polymers exhibit an additional weak but broad extension of the relaxation spectrum on the low frequency side that follows a power law, with a value between 0.5 and 0.8 for the exponent, which is due to the slow rearrangement of molecular chains known as the Rouse-Zimm type distribution, as briefly mentioned in Introduction; the spectrum being discussed here arises from density fluctuations causing a dynamic distribution in domain size.

Ngai et al. [26] assume that the relaxation spectrum is homogeneously broadened by coupling to low energy excitations. Chamberlin et al. [27], on the other hand, consider a heterogeneous distribution of exponentially relaxing domains, similar to the domain size distribution discussed here. Chamberlin’s model assumes a Gaussian distribution of domains, which results in reducing the slope of log *ε*″ vs log *Ω* at very high frequencies. The domains we consider are from the heterogeneity born of density fluctuations. Those domains with sizes that are far different from the equilibrium size, *z*_c_, have higher free energy, and are more scarce at both extremes. We apply the equipartition principle to estimate the distribution of the number *n_z_* of conformers that belong to domains of size *z*:

$$\frac{n_{z}}{n_{c}}=\exp\left[-\frac{\Delta \Psi }{kT}\left(z_{c}-z\right)\right]$$ (24)

where *n*_c_ is the number of conformers in a domain of size *z*_c_, and Δ*Ψ* is the Gibbs free energy increase when the domain size is disturbed from *z*_c_ by Δ *z* = 1.

From the Adam-Gibbs formula, Eq. (6), the formula for the perturbation in *z* from the most dominant size *z*_c_ can be obtained:

$$\frac{\tau_{z}}{\tau_{c}}=\exp\left[-\frac{\Delta \mu }{kT}\left(z_{c}-z\right)\right]$$ (25)

where *τ_c_* is the characteristic relaxation time, and *τ_z_* is the relaxation time corresponding to size *z*.

**Fig. 5:**
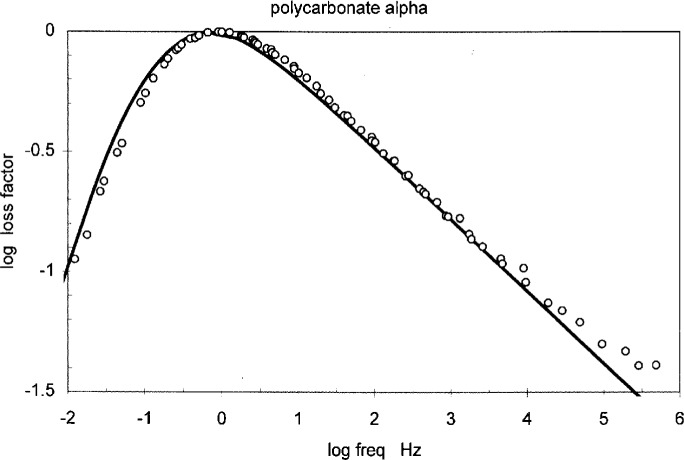
The master curve of dielectric loss spectrum for polycarbonate, obtained by shifting isothermal data.

By combining Eqs. (24) and (25), we obtain the formula for the relative number of conformers with relaxation time *τ*_z_, which is the relaxation spectrum *H*(ln *τ*):

$$H\left(\ln\tau \right)={\left(\frac{\tau }{\tau_{c}}\right)}^{\frac{\Delta \Psi }{\Delta \mu }}$$ (26)

which is a “power law” type formula, i.e., the plot of log *G*″or log *ε*″ vs log *Ω* is a straight line with the slope −Δ *Ψ*/Δ*μ*. Δ*Ψ* is the amount of free energy increase when the domain size is decreased by 1, and its value should be close to the difference between the gauche and the trans configurations, typically 4.8 kJ, so the slope is around 1/3. Dielectric data on polycarbonate obtained in the temperature range from 155 °C to 200 °C is shown in Fig. 5. This curve was constructed by shifting the individual isotherms so the loss peak is located at the origin. The slope on the high frequency side is 0.3, which would be obtained if Δ*ψ* is 4.8 kJ and Δ*μ* 14.6 kJ. The slope of similar value is observed in polyvinyl acetate and *α*-glucose. The low frequency side of the plot in Fig. 5 is steeper, about 0.5, as it is the beginning of the Rouse-Zimm spectrum. We believe, however, that the Rouse-Zimm model should apply only to polymer solutions, and that the relaxation in bulk is remote from such a model. A more appropriate model for the polymer bulk can be constructed from the distribution of strands that are dynamically active [20]. Since the shorter strands are more numerous, their contribution to the elastic energy is greater, whereas the longer strands will cause the viscosity to be greater, in proportion to the strand’ length. This results in the relationship that the relaxation time increases as the square of the strand length, while the modulus intensity is inversely proportional to the strand length, and the value of 0.5 is obtained for the slope of the spectrum on the log-log plot. Another model has been proposed by Tobolsky [28] who assumed the normal distribution for the transition zone, similar in concept to Chamberlin’s model, and obtained the slope of 0.5 for styrene-butadiene rubbers of various compositions. Tobolsky notes that stiffer chains would affect the elastic constant more severely, so that the slope could be greater. A value as high as 0.75 was found for some systems; this scheme therefore can include the Zimm value as well.

**Fig. 6:**
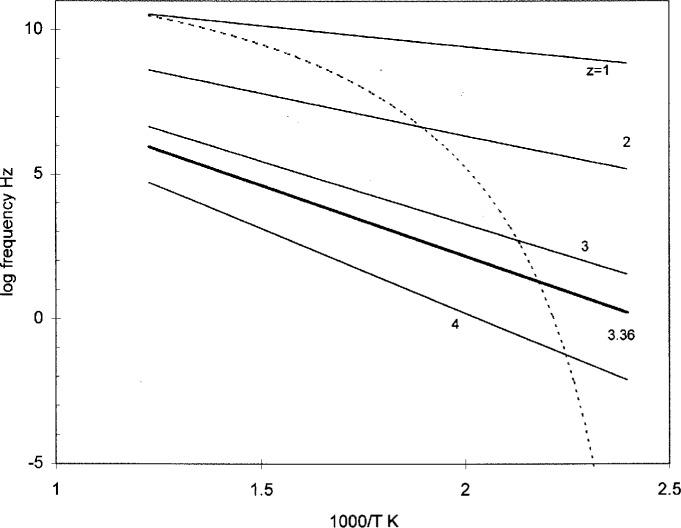
log frequency from the Vogel equation, Eq. (10), in dotted line. Five straight lines are from the Adam-Gibbs formula Eq. (6) with fixed *z*. At *T*_g_, it is estimated that *z* = 3.36.

In Fig. 6, the Vogel frequency [= 1/2 *πτ*_c_ from Eq. (10)] is plotted for the polymer with Δμ* of 14.6 kJ and *T*_0_ of 97 °C (dotted line) together with the Arrhenius plot by fixing domain sizes in the Adam-Gibbs equation for *z*, Eq. (6). Often, the slope of the Vogel line (multiplied by 2.3*R*) is quoted erroneously as the “activation energy” with values amounting to 480 kJ or more, exceeding the energy for chain scission. The derivative of the Vogel equation with respect to 1/*RT* is really the sum of the correct activation energy Δ*μ* · *z* and the term Δ*μ* · [d*z*/d(1/*RT*)]. The latter is the rate of change of the domain size with temperature; this can be a substantial quantity. A physically meaningful activation energy is the first term *z*, and this is about 96.2 kJ at 165 °C. At 147 °C, it is about 12.6 kJ, which is about the right value for viscoelastic behavior of glassy polycarbonate. The spectrum shown in Fig. 5 is a snap shot at 165 °C, and denotes the relative intensity of these Arrhenius lines at 1000/*T* ~ 2.28. The intensity for smaller domains decreases with decreasing temperature, at the expense of newly created larger domains. Below the glass transition, the size *z*_c_ becomes fixed, and the characteristic relaxation time now follows the Arrhenius line for that *z*, such as a straight line marked as 3.36, *z*_c_ at *T*_g_. The shape of the spectrum becomes markedly broader below *T*_g_ as the activation energy is different for each domain size.

## 6. The *β* Relaxation

**Fig. 7:**
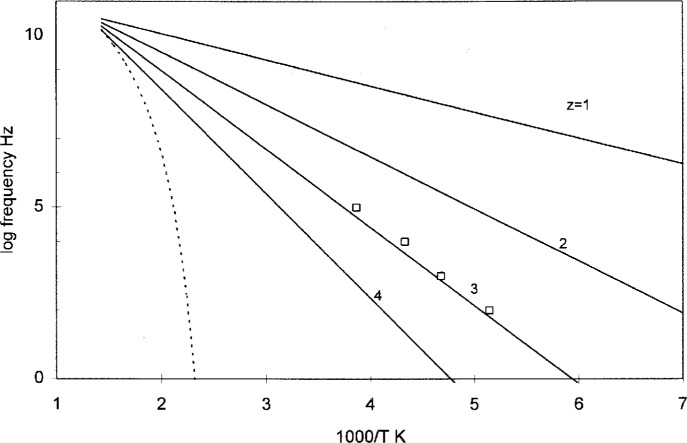
log frequency for the *β* process which follows an Arrhenius equation, Eq. (27). The dotted line is from the Vogel equation for the process as shown in Fig. 6.

The conformers within the repeat unit of a chain are typically of different sizes. When larger conformers become pinned by the neighbors (belonging to other chains) at the glass transition, there still remains a possibility for local relaxation by the smaller conformers in the same chain. As a rule the *β* relaxation requires the *intra*molecular cooperation among adjacent conformers in the same chain. The number of cooperating conformers corresponds to the number of conformers between the pinned ones. It is usually the number of conformers per repeat unit. The domain size *z_β_* for the *β* relaxation does not vary with temperature, and it is equal to the ratio of the molecular weight of the repeat unit over the average molecular weight of all conformers. This is shown in Fig. 7 for polycarbonate. The number of conformers per repeat unit is 3.3, for which *T*_g_ of 147 °C is obtained from Eq. (19). (The fraction is a result of polycarbonate containing ether linkages which requires considerably smaller activation energy.) The predicted value of *z_β_* is thus also 3.3. The activation energy, Δμ* · *z_β_*, is 46 kJ from our dielectric data, which supports the value of 3.3 for *z_β_* [20]. The formula for the *β* relaxation is given by:

$$\ln\tau_{\beta }=\ln\tau *+\frac{\Delta \mu *z_{\beta }}{k}\left(\frac{1}{T}-\frac{1}{T*}\right).$$ (27)

**Fig. 8:**
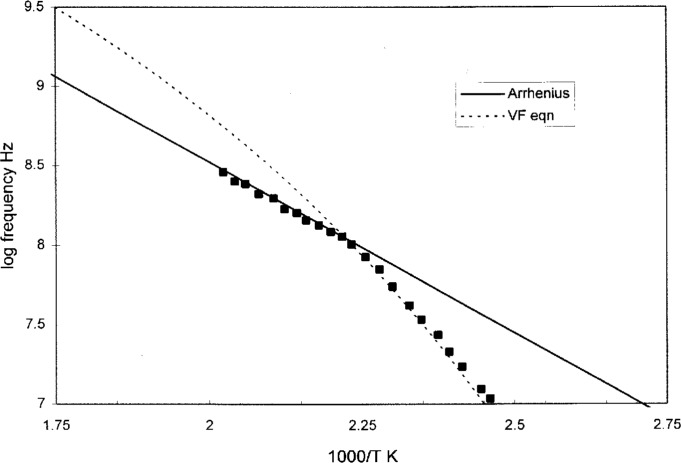
A detail of the log frequency vs 1/*T* for polyvinyl acetate in the frequency-temperature region where the *α* and *β* processes meet. Above the merging point, the process, an intramolecular cooperative relaxation process, takes over.

Here Δμ* is used as it is simply the energy barrier without need to adjust for the density that affects the intermolecular cooperativity. The intensity of the *β* relaxation is affected by the temperature and the frequency, as it depends on the population of conformers pinned in the domains. In the high temperature/frequency region where the *α* and *β* lines meet, the *β* will take over because the highest possible frequency is limited by the intramolecular cooperation even after the intermolecular relaxation has become easy. This is shown in Fig. 8 by enlarging the range of temperature/frequency where the *α* and *β* peaks merge, for polyvinyl acetate. The repeat unit in the polyvinyl acetate molecule consists of three conformers, one of which is in the branch. In this case, *z_β_* is clearly that of the number of conformers in the repeat unit, as Δμ*z_β_ is ~ 40.6 kJ and Δ*μ* * is 13.8 kJ.

## 7. Physical Aging

It is well known that the glass transition is not a thermodynamic transition between two equilibrium states, and that a glassy state is a nonequilibrium state born of too slow a rate in rearranging the molecules to reach the equilibrium state. Physical aging is a process for such a glassy state to further shift toward a state of lower enthalpy, entropy and free volume. The most curious but practically important question is the quantitative estimation of the characteristic relaxation time *τ*_c_ after the aging period *t*_a_ at a temperature *T* below the glass transition temperature.

Assuming that the aging is a slow process as compared to the temperature equilibration by thermal conduction, the intensive quantities such as pressure and temperature are considered to equilibrate quickly, say in seconds, while the domain size *z* takes a much longer time to equilibrate. Consequently, all those extensive quantities that depend on *z* will be slow to equilibrate. Thus, in the Adam-Gibbs equation, *z* and *S_z_* are time dependent while the temperature *T* is not. This makes the relaxation time *τ*_c_ also time dependent.

If the liquid is vitrified at temperature *T_f_*, the domain size thereafter becomes fixed as *z_f_*, which was the equilibrium value of *z* at *T_f_*, i.e.,

$$z_{f}=\left(\frac{T*-T_{0}}{T*}\right)\left(\frac{T_{f}}{T_{f}-T_{0}}\right).$$ (28)

Upon further cooling, *z_f_* first remains unchanged but will slowly increase with physical aging. *T_f_* is the fictive temperature which characterizes such a glassy state. *T_f_* is equal to the temperature at the intercept of the volume curve for the glassy state and the liquid state.

Substitution of *z_f_* in Eq. (28) into the first Adam-Gibbs equation, Eq. (6), will result in the equation for the nonequilibrium state:

$$\ln\tau_{f,T}=\ln\tau_{f,T*}+\frac{\Delta \mu *}{kT}\frac{T_{f}}{T_{f}-T_{0}}-\frac{\Delta \mu *}{k\left(T*-T_{0}\right)}$$ (29)

and *τ*_f,_*_T_* is the characteristic relaxation time *τ*_c_ with *T_f_* at *T*, and is time-dependent through *T_f_*. This expression has essentially been proposed by Hodge in 1986 [29], who was able to reproduce Δ*C*_p_ data for polycarbonate subjected to various thermal histories. This equation obviously reduces to the Vogel equation, when *T_f_* is equated to *T*, which is the condition for equilibrium. The Vogel equation is good only for the equilibrium condition, because the extensive and intensive thermodynamic quantities in it cannot be separated. The Doolittle free volume equation is also good only for the equilibrium condition, as both *T* and *T_f_* are imbedded inseparably in its empirical free volume parameter *f*.

Let us now introduce, for convenience, a thermodynamic variable *X_f,T_*,

$$X_{f,T}=\frac{kT}{\Delta \mu *}\frac{T_{f}-T_{0}}{T_{f}}$$ (30)

such that Eq. (29) can be written as:

$$\ln\tau =\frac{1}{X_{f,T}}+\ln\tau *-\frac{\Delta \mu *}{k\left(T*-T_{0}\right)}.$$ (31)

Unlike the Doolittle free volume *f*, this *X_f,T_* is a physically acceptable form of a function in *T_f_* and *T*. Struik’s [30] modified free volume is essentially this *X_f,T_*.

In Kovacs’ [31] well-known isothermal aging experiment the sample was initially made to equilibrate at *T*_ini_ above *T*_g_, then suddenly brought to a bath maintained at the aging temperature *T*_a_, and the volume was measured with aging time *t*_a_. Most of his experiments were conducted near *T*_g_, and the initial relaxation time is in the range of (equilibrium) *τ*_g_, on the order of tens of seconds. Not much will happen to *X_f,T_* even after 10 seconds has passed, however. There seems to be yet another induction period before *X_f,T_* begins to change. As we shall see, this is because the rate of rearranging domains is slower (by more than an order of magnitude) than the rate of viscoelastic relaxation.

Now, in all linear viscoelastic functions, in which *t* and *τ* always appear together as a single reduced variable *t*/*τ*, the relationship shown below is true:

$$\frac{d\ln\tau }{d\ln t}=1.$$ (32)

The steady state for the aging process is reached when the viscoelastic relaxation time *τ_f,T_* and aging time *t*_a_ can qualify as *τ* and *t* in Eq. (32), which can also be written as:

$$\frac{d\tau }{dt}=\frac{\tau }{t}.$$ (33)

At the beginning *t*_a_ is zero, while *τ_f,T_* has the initial value, so the initial rate of increase for *τ* would have to be ∞; *t*_a_ cannot be equal to *t* above. *τ* initially waits without changing much until *t*_a_ increases enough to satisfy Eq. (33). This is the induction period mentioned above, and it is to be distinguished from the period of temperature equilibration. If a linear viscoelastic experiments were conducted during the induction period, the data will not show the shift according to Eq. (32), i.e., one decade of elapsed time resulting in the shift of the viscoelastic data by one decade. Rather, the shift would be much less than one decade.

The induction period starts with *t*_a_<*τ_f,T_*, and dln*τ*/dln*t* < 1, until the proper ratio of *τ*/*t* as dictated by *X_f,T_* is arrived, whereupon the ratio *τ_f,T_*/*t*_a_ is maintained. For example, if the sample is subjected to an initial deep quenching, the initial *τ* is very large. Nothing will happen at first as the rearrangement of structure is extremely slow. After a long wait, *t*_a_ has elapsed sufficiently to the point Eq. (32) is satisfied with *t*_a_ as *t*. At this point, *X_f,T_*, *τ_f,T_*, *t*a, and the rate of change of *X_f,T_* all become consistent among themselves. The steady state has now been reached, and it will continue indefinitely if the aging temperature has been chosen to be below *T*_0_. If the aging temperature has instead been chosen to be above *T*_0_, then *X_f,T_* will eventually arrive at an equilibrium value where *T_f_* = *T*_a_. *τ_f,T_* will not increase further while *t*_a_ continues to increase, and Eq. (32) is again not applicable. The steady state condition exists between these two extremes, and here the nonequilibrium Adam-Gibbs formula can be applied. The kinetics of the steady state aging process can be derived by utilizing these relationships among *X_f,T_*, ln *τ*, ln *t*, etc.:

$$\begin{array}{l} \frac{1}{X_{f,T}}\frac{dX_{f,T}}{dt_{a}}=\frac{1}{X_{f,T}}\frac{dX_{f,T}}{d\tau_{f,T}}\frac{d\tau_{f,T}}{dt_{a}}=\\ -X_{f,T}\frac{d\ln\tau_{f,T}}{d\tau_{\text{f,T}}}\frac{d\tau_{f,T}}{dt_{a}}=-\frac{X_{f,T}}{t_{a}}. \end{array}$$ (34)

In deriving Eq. (34), no assumption was made to let *τ_f,T_*^−1^ be the “reaction rate constant,” i.e., we made no assumption that the apparent aging rate is the same as the rate of configurational relaxation, and in fact it is not. The solution of this differential equation is an exponential integral, and the plot of *X_f,T_* has the appearance of a stretched exponential even though a single relaxation time was assumed for the viscoelastic relaxation.

If a viscoelastic relaxation experiment is started at which point it has been aging for *t*_a_, *G*(*t*_a_) will decrease to *G*(*t*_a_)/*e* in *τ_f,T_* seconds. Reduction of *X_f,T_* at *t*_a_ to 1/*e* of this value will take much longer, approximately *τ_f,T_*/*X_f,T_. X_f,T_* is a small number, ca. 0.1 to 0.01.

We know the condition at the beginning of steady state. The initial value of *X_f,T_* requires tedious calculations, but it is not far from *X*_g_(*T*/*T*_g_), and the ratio of *τ_f,T_* to the aging time *t*_a_ is given by the formula:

$$\frac{\tau_{f,T}}{t_{a}}\approx X_{g}\frac{T}{T_{g}}\approx \frac{T}{T_{g}}\frac{k\left(T_{g}-T_{0}\right)}{\Delta \mu }.$$ (35)

Equation (35) is applicable even to the case when the initial temperature is below *T*_g_ as in the “memory effect” experiment and also most of annealing experiments where the polymer is heated to below but not too far from *T*_g_. The value of the last term is numerically close to the Doolittle free volume fraction *f*_g_, which is about 0.025. More detailed approximation yields values ranging from 0.1 to 0.01, depending on the interplay of the initial *X* and *τ*. Experimental stress relaxation data supporting *τ*/*t*_a_~*f*_g_ can be found in Ref. [20].

**Fig. 9:**
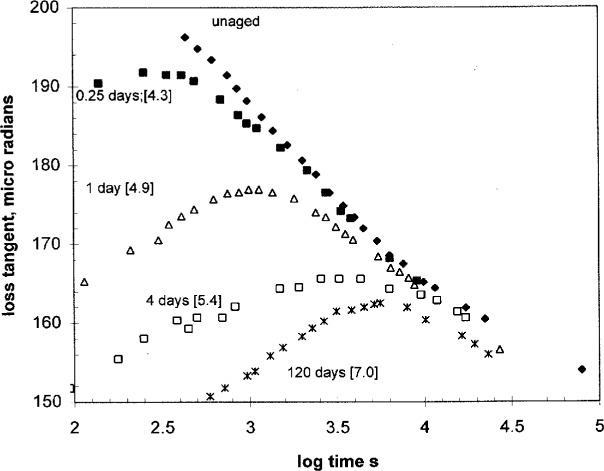
The dielectric tan *δ* for polystyrene as measured at 1 kHz at 80 °C while isothermally aged. The abscissa is the time starting at each measurement. The “days” in the figure means the aging time at 80 °C, and the number in brackets is the log(aging time in seconds).

Dielectric data for tan *δ* during isothermal aging, as measured at 1 kHz, are shown in Fig. 9. The experiment followed the procedure that: the sample is kept at 80 °C while measurements were started from time to time at various stages of aging. The time on the abscissa is the time spent for each dielectric measurement starting at the specified aging time, so the abscissa is comparable to −ln *Ω*. The dielectric tan *δ* peak is usually located at a higher frequency than for the loss peak *ε*″, so the difference between the time the peak appears and the aging time *t*_a_ should be greater than the difference between log *τ* and log *t*_a_. It is about two decades. The dielectric loss peak appears at *T*_g_ when measured at 100 Hz, and 1 kHz measurement would make little difference, ca. less than 2 °C in the temperature of the loss peak. The curve for the longest aging period (120 d) suggests that the equilibrium is approached. The relaxation time begins to stay constant while the aging time is increased indefinitely. The shortest period of 0.25 d, on the other hand, indicates the quenched condition which requires some aging time before the relaxation time begins to shift. Between these two extremes, the ratio in Eq. (32) remains nearly 1.

Another caveat for Eq. (32) has not been mentioned, and this concerns the distribution of domain sizes, which will “stretch” the spectrum. The aging process is a doubly stretched function, one due to the continuously increasing relaxation time with time, and the other due to the spreading of the spectrum due to the heterogeneity in domain sizes. The heterogeneity will cause the shape of the relaxation spectrum to change with temperature, as the apparent activation energy is proportional to the domain size. The shape will become flatter at lower temperatures. Visco*plastic* data from the stress-strain curves of glassy polymers support the reduction of the power law exponent in the high frequency range as the temperature is lowered well below *T*_g_ [20].

## 8. Summary

The Vogel equation can be derived from the temperature dependence of the domain size for intermolecular cooperativity from the Adam-Gibbs formula in equilibrium. The measured entropy approaches zero at a temperature *T*_0_ that depends on the conformer size, though the molecules are still in the highly amorphous disordered state, i.e., high configurational entropy. In the cooperativity model, all conformers within a domain are allowed to relax in unison. The internal degree of freedom within a domain is reduced to that of one conformer. As the measurable entropy is the configurational entropy per mole of conformers, it decreases more rapidly as the domain grows, than the conformational entropy that describes disorder in polymer molecules.

The heterogeneity of domain sizes are considered to be responsible for the distribution of relaxation times, as manifest in Kovacs’ memory effect and Hodge’s sub-*T*_g_ relaxation. A power law *τ*^−n^ is derived for the relaxation spectrum based on the free energy of domain size fluctuations.

The van der Waals excess free volume is much greater than the Doolittle free volume. The latter is evaluated from the relaxation data, and its expansion coefficient, *α*_f_ is not equal to Δ*α*, but consistently differs depending on the molecular size. From the size of the conformer, *T*_g_ can be estimated. For a single conformer to be able to relax free of the neighbors interference, it needs the free volume comparable to the occupied volume. It has been shown that the coefficient of thermal expansion for the free volume fraction is approximately equal to the ratio between the *kT* and Δμ*, the latter being the energy barrier for the bond rotation. The amount of free volume trapped at *T*_g_ is greater for the glass vitrified at a higher temperature, while its glassy modulus is lower. This is true for crosslinked polymers as well.

A relationship has been derived relating the ongoing relaxation time to the aging time. The ratio of the characteristic relaxation time to the elapsed aging time is equal to the quantity *kT*/Δ*μ*z, which is in the range of 0.1 to 0.01 depending on the temperature and thermal history of the glassy state being aged.
